# Prevalence of depression and anxiety, and their relationship to social support among patients and family caregivers of rare bone diseases

**DOI:** 10.1186/s13023-022-02611-3

**Published:** 2023-01-26

**Authors:** Xuefeng Lai, Yuling Jiang, Yue Sun, Zhijun Zhang, Shengfeng Wang

**Affiliations:** 1 Department of Epidemiology and Biostatistics, School of Public Health, Peking University, 38 Xueyuan Road, Haidian District, Beijing, 100191 China; 2 School of Public Health, Peking University, 38 Xueyuan Road, Haidian District, Beijing, 100191 China; 3 Lingyi Foundation for Rare Bone Diseases, 1A801, Gaofa West Bund Garden, 5th Avenue, Anbao District, Shenzhen, 518133 China

**Keywords:** Rare bone diseases, Depression, Anxiety, Social support

## Abstract

**Background:**

Rare bone diseases (RBDs) are a set of inherited rare diseases that can cause disability and have a devastating impact on families affected, which may lead to a particular high prevalence of psychological disorders in patients and caregivers. Social support plays a role in the well-being of families with rare disease patients, but its effect on psychology of RBD families remains unclear. The purpose of the current cross-sectional quantitative study was to investigate the frequency of depression and anxiety, and their relationship with social support among RBD patients and family caregivers.

**Results:**

A total of 196 participants responded to the questionnaire, including 72 patients and 124 caregivers. Depression was detected among 33.8% of patients and 57% of caregivers, and anxiety disorder was presented in 28.6% of patients and 50% of caregivers. Higher depression scores and anxiety scores were found in both patients and caregivers with an education level of ≤ middle school and monthly income of ≤ ￥2000 (all *P* < 0.05). The mean (SD) scores of Social Support Rating Scales in patients and caregivers were 37.06 (8.05) and 38.31 (5.76), respectively. After adjusting for gender, age, monthly income, education, employment and marital status, the reverse associations between depression scores, anxiety scores and social support were found merely in caregivers (depression & social support: *β* = − 0. 46, *P* < 0.001, anxiety & social support: *β* = − 0. 44, *P* < 0.001), specifically for subjective support (depression & subjective support: *β* = − 0.94, *P* < 0.001, anxiety & subjective support: *β* = − 0.87, *P* < 0.001).

**Conclusions:**

The study identified a high prevalence of depression and anxiety among RBD patients and caregivers, and pointed out the significance of social support in alleviating psychological distress. In order to provide RBD families with comprehensive assistance, the government should actively develop programs aimed at psychological aid, policy advocacy and tangible support.

## Background

Rare Bone Diseases (RBDs) are a group of rare genetic disorders with almost 400 different forms involving the skeletal system with characteristic clinical and radiographic features [1]. RBDs account for 5% of all birth defects, and are important causes of disability worldwide [2]. Many individuals with genetic RBD may suffer from significant physical disabilities [3] and require periodic medical follow up, corrective surgery, drug therapy and physiotherapy, as well as specific daily care practices [4]. Because of the high cost, long course and incurable nature of the disease, patients with a rare disease often experience social difficulties and psychological stress besides physical health challenges [5]. Moreover, as they are often avoided, misunderstood and blamed due to people’s lack of awareness [5], the mental health of this population might deteriorate further. Timely recognition of RBD patients’ distress, followed by appropriate and targeted support is crucial to improve outcomes of patients [6].

Apart from personal experience of suffering, RBD is also a family event. As the case with the most chronic conditions, RBD brings both sustaining financial and emotional burden to the family, especially the members who care for the patients [7]. Since the adverse psychological conditions of caregivers directly affect the mental and physical health of patients [7], detecting and addressing psychological distress of caregivers are important to ensure the quality of family care they can provide. Given that social support may have a positive impact on psychological adjustment [6], it is critical to assess the conditions and needs of social support, so as to providing effective coping strategies for families with RBD patient.

Currently, some studies have been conducted to characterize the psychological status on RBD patients and family caregivers, however, most of which were interviews or qualitative analyses of a small sample [8–11]. Although there were several quantitative studies indicating a high level of emotional burden in these groups [4, 12–15], the sample size was still undesirable (< 100). While results from a study with 184 participants showed that mental performance of RBD patients was similar to the standard values [16]. To our knowledge, however, most of previous quantitative analyses evaluated merely the overall mental condition. Prevalence of depression and anxiety, the two most common mental problems [17], remains unclear in this population. Additionally, given the role of social support on alleviating psychological distress [18], published studies have involved social wellbeing of RBD families [13, 14, 19], yet none of which specifically described the impact of social support on psychological status of RBD patients and caregivers.

## Methods

### Aims and design

Based on the considerations above, objectives of the cross-sectional survey were to describe the frequency of depression and anxiety and the level of social support, in addition, to understand the relationship between psychological conditions and social support on RBD patients and caregivers.

### Participants

By the assistance of Lingyi Rare Bone Diseases Care Center in Shenzhen, 196 potential participants, including 72 adult patients and 124 caregivers of children with RBD were recruited to finish the electronic questionnaire via WaChat, which is a widely used social media platform in China. Only those who signed the informed consent participated in this survey in August 2021. All the participants were informed that data were collected and analyzed anonymously and they could quit the questionnaire at any time. Ethics approval was given through the Institutional Ethics Committee of Peking University (IRB00001052-21075).

### Assessment instruments

Demographic information was collected pertaining to age, gender, household income, education attainment, employment, marital status, caregiver’s relation with the patient, patient’s course of disease (years) and type of RBD, the care condition of patients (home care or medical institutionalized care).

Depression was measured by the Patient Health Questionnaire-9 (PHQ-9), a 9-item screening tool to determine the severity of depression symptoms over the last 2 weeks [20]. The total score of PHQ-9 ranges from 0 to 27, and scores of 5–9, 10–14, 15–19 and ≥ 20 present mild, moderate, moderately severe and severe depression, respectively. A score of 10 or above is recommended as a single cut-off point for major depression [21]. As the most reliable screening tool for depression, PHQ-9 has demonstrated adequate reliability and validity and has been used in different populations from health care and community settings, including patients with rare disease [22–24].

General Anxiety Disorder Scale-7 (GAD-7), a screening tool for anxiety, was used to measure the frequency of anxiety symptoms over the past 2 weeks [25]. Each item of the scale describes one of the typical anxiety symptoms. The total score ranges from 0–21, and scores of 5–9, 10–14 and ≥ 15 present mild, moderate and severe anxiety, respectively, with a total score of ≥ 10 identified as ideal cut-off point indicating generalized anxiety disorder [26]. With favorable reliability, high sensitivity and specificity for screening GAD [26], the scale has been used in both general population [24] and patients [18].

The level of social support was evaluated with Social Support Rating Scale (SSRS), which has good reliability and validity [27] and is the most prevalent questionnaire for measuring social support of various population over 14 years old in China [28, 29]. SSRS contains 10 items in 3 subscales: subjective social support, objective social support and the utilization of social support [27], with a total score of 66. Overall, higher scores indicate greater levels of individual social support. The subjective support score of 8–24 is defined as low, 25–32 as high; the objective support score of 1–13 is defined as low, 14–22 as high; the score of 3–9 in utilization of support is defined as low, 10–12 as high [30, 31]. Apart from SSRS, respondents were also asked to answer to the following questions: (i) What aspects of support and aid would you like to obtain? (ii) What kind of financial support did you receive during the treatment?

As a result, 187 individuals completed all the three scales, with 192, 194 and 193 respondents completing PHQ-9, GAD-7 and SSRS, respectively.

### Statistical analysis

Mean and standard deviations (SD) were calculated for quantitative demographic characteristics, the scores of the PHQ-9, GAD-7 and SSRS. Categorical variables, along with the rates of anxiety (GAD score ≥ 10) and depression (PHQ-9 score ≥ 10), were reported using frequencies. One-way ANOVA or chi-squared test was used for independent samples to investigate differences between subgroups in depression, anxiety and social support. The relations between depression, anxiety and social support were calculated applying linear regression. Statistical analyses were conducted using R.4.4.1, and a *P* value < 0.05 (two-tailed test) denotes statistical significance.

## Results

### Demographic characteristics

A sample of 196 individuals was analyzed, including 72 adult patients and 124 caregivers, with a median age of 24.50 (IQR: [20.00, 33.00]) and 37.00 (IQR: [32.75, 42.00]), respectively. There were 46 (63.9%) female patients and 108 (87.1%) female caregivers. Nearly half of the respondents were of middle school education or below (51.4% for patients, 47.6% for caregivers), and most of the respondents were not having a fulltime job (66.7% for patients, 58.1% for caregivers) and had household income of ￥5000 or below (68.0% for patients, 76.6% for caregivers). The majority disease of those participated was osteogenesis imperfecta (OI, 83.3%), X-linked hypophosphate rickets (9.7%), achondroplasia (1.4%) and other RBDs accounted for less than 20% of the patients (Table 1). For patients’ care status, 98.98% of the 196 RBD families were cared at home, while only 2 (1.02%) patients opted for institutionalized care in rehabilitation center.

**Table 1:** Demographic characteristics of the respondents

	Patients	Caregivers
n	72	124
Gender, n (%)		
Male	26 (36.1)	16 (12.9)
Female	46 (63.9)	108 (87.1)
Age, median [IQR]	24.50 [20.00, 33.00]	37.00 [32.75, 42.00]
Education, n (%)		
≤ Middle school	37 (51.4)	59 (47.6)
Senior high school	10 (13.9)	22 (17.7)
≥ Bachelor's degree	25 (34.7)	43 (34.7)
Monthly income, n (%)		
≤ ￥2000	24 (33.3)	31 (25.0)
￥2000–5000	25 (34.7)	64 (51.6)
≥ ￥5000	23 (31.9)	29 (23.4)
Employment, n (%)		
Employed	24 (33.3)	52 (41.9)
Unemployed	48 (66.7)	72 (58.1)
Course of disease, median year [IQR]	23.00 [18.00, 32.25]	7.00 [4.00, 12.00]
Disease types, n (%)		
Osteogenesis imperfecta	60 (83.3)	77 (62.1)
X- linked hypophosphate rickets	7 (9.7)	10 (8.1)
Achondroplasia	1 (1.4)	19 (15.3)
Others	4 (5.6)	18(14.5)
Marital status, n (%)		
Married	22 (30.6)	116 (93.5)
Not married	50 (69.4)	8 (6.5)
Patient’s care status, n (%)		
Institutionalized care	0 (0)	2 (1.61)
Family care	72 (100.00)	122 (98.39)

### Depressive and anxiety symptoms

The mean (SD) PHQ-9 score were 8.18 (6.29) in patients and 11.60 (7.22) in caregivers, with 24 (33.8%) patients and 69 (57%) caregivers showed score of ≥ 10, indicating moderate, moderately severe or severe depression levels. Regarding anxiety symptoms, the mean (SD) of GAD-7 scores were 6.89 (5.76) in patients and 10.50 (6.62) in caregivers, with 20 (28.6%) patients and 62 (50.0%) caregivers had a score of ≥ 10, presenting moderate or severe anxiety disorder. The scores of depression symptoms (*P* = 0.001) and anxiety (*P* < 0.001) in caregivers were higher than those in patients (Table 2).

**Table 2:** PHQ-9, GAD-7 and SSRS scores of RBD patients and caregivers

	Patients	Caregivers	*P* value
n of PHQ-9	71	121	–
PHQ-9 scores, mean (SD)	8.18 (6.29)	11.60 (7.22)	0.001^a^
Depression, n (%)			0.003^b^
No	25 (35.2)	24 (19.8)	–
Mild	22 (31.0)	28 (23.1)	–
Moderate	11 (15.5)	19 (15.7)	–
Moderately severe	8 (11.3)	32 (26.4)	–
Severe	5 (7.0)	18 (14.9)	–
PHQ-9 score ≥ 10, n (%)	24 (33.8)	69 (57.0)	0.003^c^
n of GAD-7	70	124	–
GAD-7 scores, mean (SD)	6.89 (5.76)	10.50 (6.62)	< 0.001^a^
Anxiety, n (%)			0.006^b^
No	31 (44.3)	26 (21.0)	–
Mild	19 (27.1)	36 (29.0)	–
Moderate	10 (14.3)	21 (16.9)	–
Severe	10 (14.3)	41 (33.1)	–
GAD-7 Score ≥ 10, n (%)	20 (28.6)	62 (50.0)	0.006^c^
n of SSRS	72	121	–
SSRS sum, mean (SD)	37.06 (8.05)	38.31 (5.76)	0.208^a^
Utilization of support, mean (SD)	6.32 (1.85)	6.35 (1.72)	0.917^a^
Objective support, mean (SD)	8.08 (3.33)	8.28 (2.80)	0.659^a^
Subjective support, mean (SD)	22.65 (5.05)	23.69 (3.26)	0.086^a^

^a^*P* value of Student’s t test for difference of scores between patients and caregivers

^b^*P* value of one-way ANOVA for distribution of depression and anxiety levels in patients and caregivers

^c^*P* value of chi square test for distribution of depression and anxiety levels in patients and caregivers

For patients, higher depression scores were found in lower education levels (*P* = 0.003), and higher anxiety scores were found in female (*P* = 0.038) and those with less education (*P* = 0.007). Regarding caregivers, higher depression scores were found in those with less education (*P* = 0.002), lower household income (*P* = 0.007), and unemployment (*P* = 0.043); and higher anxiety scores were found in those with less education (*P* = 0.022) and lower household income (*P* = 0.018, Table 3).

**Table 3:** Subgroup analysis of depression and anxiety of RBD patients and caregivers

Factors	Depression scores, mean (SD)		Anxiety scores, mean (SD)	
	Patients (n = 71)	Caregivers (n = 121)	Patients (n = 71)	Caregivers (n = 121)
Gender				
Male	6.77(5.98)	11.88(8.48)	5.04(5.09)	11.00(8.03)
Female	9.00(6.39)	11.55(7.05)	7.98(5.91)	10.43(6.43)
*P*-value	0.151	0.868	0.038	0.748
Education status				
≤ Middle school	10.42(6.32)	13.76(6.98)	8.97(6.00)	12.07(6.55)
Senior high school	6.30(4.95)	9.91(7.75)	4.00(3.62)	8.95(6.92)
≥ Bachelor's degree	5.72(5.72)	9.44(6.48)	5.12(5.15)	9.14(6.12)
*P*-value	0.003	0.002	0.007	0.022
Monthly income				
≤ ￥2000	10.13(5.35)	14.32(6.54)	8.18(5.18)	12.45(6.26)
￥2000–5000	7.52(6.48)	11.24(7.47)	6.00(5.75)	10.50(6.97)
≥ ￥5000	6.82(6.77)	9.30(6.57)	6.61(6.31)	8.41(5.70)
*P*-value	0.072	0.007	0.317	0.018
Employment status				
Employed	6.71(6.72)	10.02(6.80)	5.83(5.64)	9.29(6.57)
Unemployed	8.94(5.99)	12.70(7.34)	7.40(5.81)	11.38(6.56)
*P*-value	0.159	0.043	0.285	0.083
Marital status				
Married	9.73(6.76)	11.44(7.11)	7.40(5.87)	10.33(6.53)
Not married	7.49(6.01)	13.75(8.81)	6.68(5.77)	13.00(7.82)
*P*-value	0.167	0.384	0.64	0.217

### Social support and its relationship with psychological distress

The mean (SD) scores of SSRS in patients and caregivers were 37.06 (8.05) and 38.31 (5.76), respectively. There was no difference between patients and caregivers in total scores and sub-scale scores of SSRS (Table 2).

After adjusting for age, gender, education attainment, monthly income, employment status and marital status, the reverse associations between PHQ scores (β = − 0.46, *P* < 0.001), GAD scores (β = − 0.44, *P* < 0.001) and social support were found in caregivers, specifically for subjective support (PHQ: β = − 0.94, *P* < 0.001, GAD: β = − 0.87, *P* < 0.001). Nevertheless, there was no significant effects of social support to GAD and PHQ scores in patients (Table 4).

**Table 4:** Adjusted linear regression for predicting GAD and PHQ with social support in RBD patients and caregivers

Social support	PHQ-9			GAD-7		
	β	95% CI	*P*-value	β	95% CI	*P*-value
Patients^*^						
Total	− 0.22	− 0.44, 0.001	0.051	− 0.16	− 0.36, 0.03	0.103
Objective	− 0.28	− 0.84, 0.28	0.319	0.02	− 0.48, 0.52	0.946
Subjective	− 0.29	− 0.61, 0.02	0.065	− 0.27	− 0.55, 0.01	0.055
Utilization	− 0.57	− 1.55, 0.41	0.252	− 0.63	− 1.50, 0.24	0.154
Caregivers^*^						
Total	− 0.46	− 0.67, − 0.24	< 0.001	− 0.44	− 0.63, − 0.24	< 0.001
Objective	− 0.94	− 1.38, − 0.51	< 0.001	− 0.87	− 1.27, − 0.47	< 0.001
Subjective	− 0.44	− 0.85, − 0.03	0.030	− 0.52	− 0.90, − 0.15	0.006
Utilization	− 1.06	− 1.80, − 0.32	0.010	− 0.77	− 1.46, − 0.09	0.030

^*^Adjusted with sex, age, education level, monthly income, employment status and marital status

### Needs of families with RBD patients

**Fig. 1:**
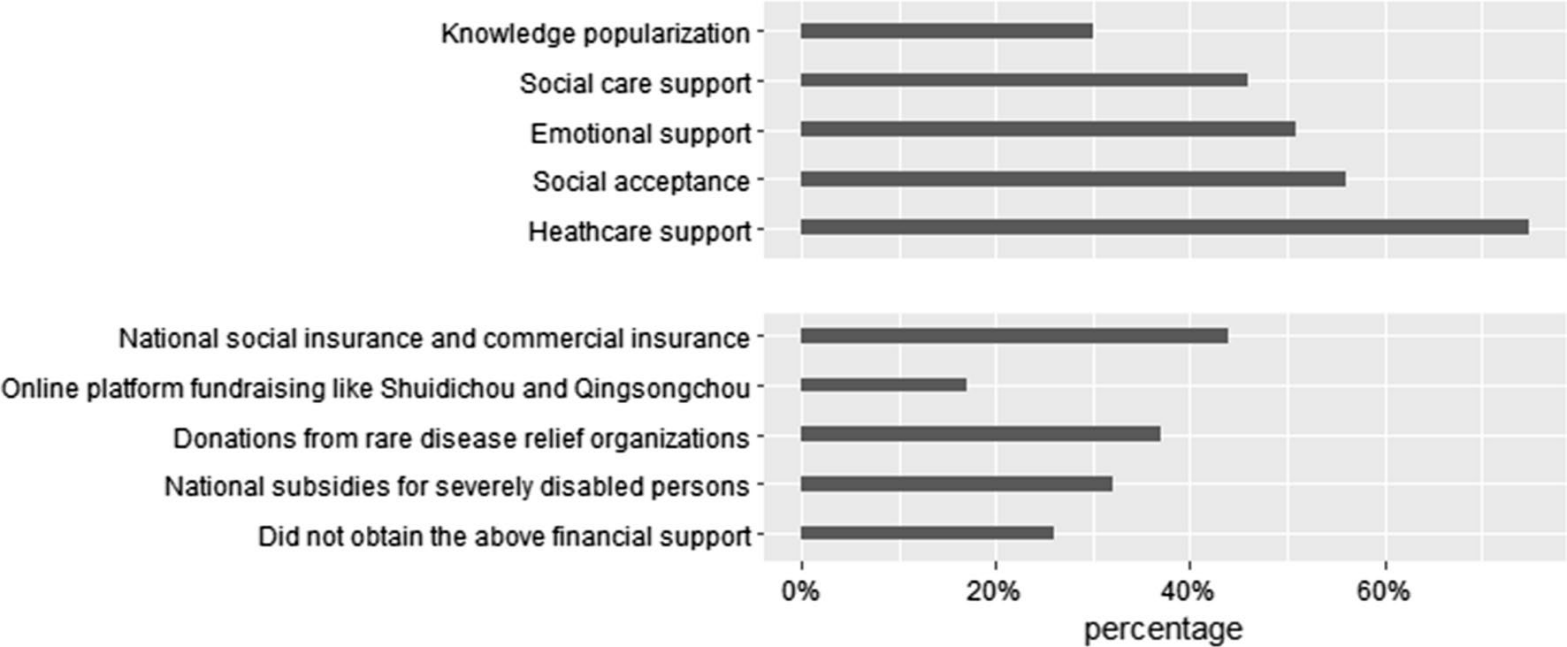
Proportion of patient needs and financial support that respondents have received. Knowledge popularization: Such as timely understanding of the disease related medical care knowledge and policy information guidance, to assist in the development of disease course planning. Social care support: Such as implement the policy of subsidizing nursing fees and reducing the fees charged by professional nursing institutions. Emotional support: Such as activities, mutual talks and encouragements between patients' organizations. Social acceptance: Such as popularizing the knowledge of rare bone diseases to the public so as to gain the acceptance and respect of patients with rare bone diseases and reducing discrimination. Healthcare support: Such as increasing subsidies for medical expenses, formulating a scientific and effective treatment plan

According to the results of multiple choices questions, about 75% of the respondents put forward the needs of healthcare support (such as increasing subsidies for medical expenses, formulating a scientific and effective treatment plan), while only 30% respondents asked for RBD knowledge popularizing (Fig. 1). Moreover, regarding financial support that respondents have received, national social insurance and commercial insurance were the most common support. However, 26% of patients or caregivers did not receive any kinds of financial support listed (online fundraising, national subsidies for disability, national social insurance or commercial insurance, and donations from philanthropic organizations, Fig. 1).

## Discussion

The study was one of the first to investigate the depression, anxiety, and their association with social support on RBD patients and family caregivers using questionnaire design. The findings indicated the high prevalence of depression and anxiety, and relatively poor social support in RBD patients and family caregivers. Moreover, the results showed the significant reverse correlation between social support and depressive symptoms or anxiety, and described the urgent needs of families with RBD patient for healthcare support, providing insights into the form and content of targeted support to this group of people.

As expected, the results reported higher prevalence of depression and anxiety in RBD patients than norm samples [32], while a lower rate of major depression (33.8% vs. 42%) and a higher rate of generalized anxiety disorder (28.6% vs. 23%) than Uhlenbusch’s study in rare diseases [22]. Given the generally belief that females were more vulnerable to depression and anxiety than males [33], different results of the two surveys can be in some extent due to an overwhelming proportion of women in the previous study. Moreover, higher scores in GAD-7 were found in our study, indicated that patients with RBD might experience a higher degree of anxiety than patients with other rare diseases listed in Uhlenbusch’s study (most of which were non-disabling). Regarding psychology of caregivers, our study reported higher prevalence than existing studies on OI or other chronic diseases in both depressive symptoms [12, 34, 35] and anxiety [34, 35], despite the variety of tools for depression or anxiety measurement. These findings highlighted the severe psychological distress of families with RBD patient, which can be partially attributed to the disability or visible abnormity of growth and development caused by RBD [36], along with the sequential experiences of stigmatization and discrimination [37]. Notably, the results that caregivers had significantly severe depression symptom and anxiety than patients seemed surprising but were consistent with a published study on other chronic disease [38]. Apart from the small sample of patients, these results can be partially explained by the fact that the majority of family caregivers were parents, who were extremely concerned about the physical conditions of their children and had to bear tremendous burden (such as difficulty in social life, significant time cost and financial burden) [39], suggesting the great needs of caregivers for mental interventions such as aid of psychological counseling and support from other caregivers.

Our results showed that depressive symptoms and anxiety of caregivers corelated with the level of social support inversely, especially in objective support. This was generally consistent with previous studies on other rare disease [18, 40], adding to evidence that improving social support is significant for families with RBD patient to cope with emotional distress. This may in some extent explain why elevated depression and anxiety of caregivers related to unemployment. Besides, our results indicated that nearly all RBD patients have the needs of home care. With the progression of disease, family caregivers are likely to give up their full-time job and make concession for patient care since time cost of caring for a patient with disability is significant [41]. Given that lack of social support is a crucial determinant of depression [42], involuntary unemployment might decrease the social support [43] and consequently aggravate depression symptoms. Notably, social support scores of RBD patients were lower than those of patients with other chronic diseases [18, 44], whereas the social support scores of RBD caregivers were higher than those reported in previous studies [45]. This may not be surprising since patients with RBD have lower level of activity and participation in employment [19] due to the long course and high deformity rate of disease [3]. Instead, compared with caregivers of children with other chronic disease, families of children with rare disease are more likely to connect well with patient groups, leading to a higher level of social support [46]. Nevertheless, the results showed that both RBD patients and caregivers geared low level of social support. This was in line with previous studies reported that many caregivers with OI children had family relationships deteriorated or received no social support [4, 8], highlighting the necessity to help families of RBD cope with these problems effectively and increase their adjustment to the disease. In this regard, attending patient groups or care centers, which have been confirmed to be one of the most effective ways [39], is highly recommended for RBD families to obtain social support by sharing information of the disease, difficult conditions experienced, and ways to cope with these availably.

The study identified that healthcare support was most needed for families with RBD patient. This was in line with Joldic’s study, which reported the promotion of policy on medical insurance and information related to treatment to be the greatest needs for families of patient with rare disease [47]. However, the need for knowledge popularization was raised by 30% of the respondents in this study, which was far less than Arabaci’s survey [8]. The results were not surprising since currently patients and caregivers are able to obtain health information online easily because of the rapid development of the Internet [48]. Besides, due to the fact that the population in this study were recruited from a patient organization, respondents had better access to relevant information, resulting in reduced demand of knowledge [49]. Additionally, social acceptance and emotional support were also highly valued by respondents, consistent with the conclusion of previous studies that suffering discrimination [50] and caregivers’ lack of psycho-education [8] were the major challenges for patients with rare diseases. These findings indicated the importance of government efforts to widely publicize the knowledge on RBD, so as to improve the public's acceptance of patients and eliminate discrimination. Meanwhile, relevant institutions should work towards solving these issues by facilitating the implementation of National Strategy and Action Plan for Rare Diseases and supporting society organizations to fulfill the needs of RBD families.

The current quantitative study provides a first insight into depression, anxiety and their association with social support in RBD patients and caregivers. However, this study also has several limitations. There may be potential selective bias since the participants were allowed to withdraw from the questionnaire at any time. However, the bias is negligible as both patients and caregivers had response rates of more than 97%. Additionally, this study did not cover all categories of RBD. Though the disastrous consequences and burden caused by RBD are similar due to the shared pathophysiologic steps of bone metabolism [51], different RBD are heterogeneous in aetiology, in their onset and severity [52]. Previous study on OI reported significant association between lower patient physical functioning and higher caregiver stress [12]. However, no studies have investigated whether and how RBDs with different morbidity and mortality may lead to distinct psychological effects on patients and caregivers. Further studies should focus on this topic and preferably include more RBD families of various disease categories to improve generalizability of the results. Besides, our study lacked detailed data to distinguish the severity of RBD, which might affect the mental health of respondents and should be considered for further research.

## Conclusion

The study is the first quantitative study to identify depression and anxiety, and their relationship with social support on RBD patients and caregivers. The data show a high prevalence of depression and anxiety, and a relatively low level of social support on families with RBD patient. Efforts from government and public institutions are needed to strengthen social awareness and promote the implementation of policy on RBD. Furthermore, patient organizations should be involved to understand the issues of families with RBD patient and disseminate relevant information among the RBD community, so as to adequately address their needs and help to cope with the disease.

## Data Availability

The datasets used and/or analyzed during the current study are available from the corresponding author on reasonable request.

## Abbreviations

RBDs: Rare bone diseases
PHQ-9: Patient health questionnaire-9
GAD-7: General anxiety disorder scale-7
SSRS: Social support rating scale
OI: osteogenesis imperfecta
